# An easily accessible sulfated saccharide mimetic inhibits in vitro human tumor cell adhesion and angiogenesis of vascular endothelial cells

**DOI:** 10.3762/bjoc.8.89

**Published:** 2012-05-29

**Authors:** Grazia Marano, Claas Gronewold, Martin Frank, Anette Merling, Christian Kliem, Sandra Sauer, Manfred Wiessler, Eva Frei, Reinhard Schwartz-Albiez

**Affiliations:** 1 German Cancer Research Center (DKFZ), Im Neuenheimer Feld 280, 69120 Heidelberg, Germany; 2 Calvatis GmbH, Dr.-Albert-Reimann-Str. 16a, 68526 Ladenburg, Germany; 3 Kao Germany GmbH, Pfungstädter Str. 92–100, 64297 Darmstadt/Eberstadt, Germany,; 4 Biognos AB, Generatorsgatan 1, 41705 Goeteborg, Sweden

**Keywords:** angiogenesis, biomimetic synthesis, carbohydrates, in silico blind docking, melanoma cells

## Abstract

Oligosaccharides aberrantly expressed on tumor cells influence processes such as cell adhesion and modulation of the cell’s microenvironment resulting in an increased malignancy. Schmidt’s imidate strategy offers an effective method to synthesize libraries of various oligosaccharide mimetics. With the aim to perturb interactions of tumor cells with extracellular matrix proteins and host cells, molecules with 3,4-bis(hydroxymethyl)furan as core structure were synthesized and screened in biological assays for their abilities to interfere in cell adhesion and other steps of the metastatic cascade, such as tumor-induced angiogenesis.

The most active compound, (4-{[(β-D-galactopyranosyl)oxy]methyl}furan-3-yl)methyl hydrogen sulfate (GSF), inhibited the activation of matrix-metalloproteinase-2 (MMP-2) as well as migration of the human melanoma cells of the lines WM-115 and WM-266-4 in a two-dimensional migration assay. GSF inhibited completely the adhesion of WM-115 cells to the extracellular matrix (ECM) proteins, fibrinogen and fibronectin.

In an in vitro angiogenesis assay with human endothelial cells, GSF very effectively inhibited endothelial tubule formation and sprouting of blood vessels, as well as the adhesion of endothelial cells to ECM proteins. GSF was not cytotoxic at biologically active concentrations; neither were 3,4-bis{[(β-D-galactopyranosyl)oxy]methyl}furan (BGF) nor methyl β-D-galactopyranoside nor 3,4-bis(hydroxymethyl)furan, which were used as controls, eliciting comparable biological activity. In silico modeling experiments, in which binding of GSF to the extracellular domain of the integrin α_v_β_3_ was determined, revealed specific docking of GSF to the same binding site as the natural peptidic ligands of this integrin. The sulfate in the molecule coordinated with one manganese ion in the binding site.

These studies show that this chemically easily accessible molecule GSF, synthesized in three steps from 3,4-bis(hydroxymethyl)furan and benzoylated galactose imidate, is nontoxic and antagonizes cell physiological processes in vitro that are important for the dissemination and growth of tumor cells in vivo.

## Introduction

Adhesion of mammalian cells to the extracellular matrix (ECM) is mediated by protein–protein and protein–carbohydrate interactions. Alterations in the expression of cell-surface molecules lead to the dissemination of metastatic cells from tumor tissue [1–2]. Cell surface molecules in melanoma, which are important for their metastatic property, have been intensely investigated. Among these, integrins, which are hetero-dimeric integral membrane proteins, are involved in protein–protein mediated adhesion of cells to the extracellular matrix (ECM).

Aberrant levels of several integrins have been observed in malignant melanomas. While some integrins such as α_6_β_1_ and α_v_β_5_ are down-regulated, others such as α_v_β_3_, α_4_β_1_ and α_3_β_1_ are up-regulated [3]. Integrins bind to ECM-proteins and this constitutes the strongest interaction in the adhesion processes. The importance of the fibrinogen receptor α_v_β_3_ in malignancy is well described for melanomas [4]. It is also a prerequisite for the activation of pro-MMP-2, a secreted metalloprotease important for cell migration through the basal layer [5].

Due to the vast variability of branched saccharide chains, more specific interactions between cells and the ECM and among cells are mediated by binding of proteins such as lectins to oligosaccharides. Upon progression to higher malignancy, the glycosylation patterns of glycoproteins and glycosphingolipids on tumor cell surfaces undergo several alterations [6]. These changes are closely associated with distinct cellular processes, such as adhesion to the ECM, and modulation of the tumor-associated microenvironment, which represent advantages for the capacity of the cell to invade the host tissue and to secure undisturbed growth [7]. The glycosylation pattern of tumor cells is therefore the focus in the development not only of new tools for tumor diagnosis and monitoring but also in the design of new anticancer drugs [8]. Attempts have been made to mount an immune response against tumor oligosaccharides by carbohydrate vaccines [9] and also to inhibit adhesive processes, such as the interaction between oligosaccharides and lectins, by synthetic carbohydrate analogues [10–13].

Based on Schmidt’s imidate strategy [14], we have developed a method for the synthesis of a library of saccharide-mimetics containing furans. Furan, especially as its bis-hydroxymethylated derivative, was chosen as a core molecule because it mimics a furanose but without an optically active center, making the synthesis of defined molecules much easier than on a furanose core. Synthetically it is easily accessible and is a dienophile, which allows attachment of marker molecules in a Diels–Alder reaction, leaving the hydroxy groups of the carbohydrate moieties unaffected [15]. With biotin-labeled Diels–Alder products of branched saccharide mimetics, discrete staining patterns were observed on surfaces of human epithelial tumor cells, but not on immortalized normal fibroblasts. Screening of the library to find members with anti-adhesive properties showed that 3,4-bis{[(β-D-galactopyranosyl)oxy]methyl}furan (BGF) could inhibit the adhesion of murine B16F10 melanoma cells to several ECM-proteins [15].

Probably, more important than uncharged saccharides are carbohydrates that contain acidic residues, such as sialic acids or sulfated saccharides. Glycosaminoglycans (GAG) are long polysaccharide chains containing sulfated saccharides of uronic acids (either iduronic or glucuronic acid) and glucosamine or galactosamine as repetitive disaccharide units. GAGs exist at the cell-surface as well as in the ECM and are attached to proteins [16]. Furthermore, the overexpression of the charged blood group antigen sialyl Lewis^x^ consisting of the terminal NeuNAcα2-3Galβ1-4(Fucα1-3)GlcNAc-group is correlated with carcinogenesis. It is recognized and bound by selectins, which are a subgroup of lectins that play an important role as cell-surface molecules [17]. Molecular dynamics simulations have shown that furans with two saccharides bound to hydroxymethyl groups show a nearly perfect alignment with each of the three terminal saccharides in Lewis^y^ [15], another member of the Lewis histo blood group family, which is involved in tumor cell adhesion [18] as well as in tumor-induced angiogenesis [19]. To further develop the molecules found to be biologically active in our earlier study, and because charged saccharides are important in tumor cell interactions, we decided to include a charged pharmacophor. Here, we describe the synthesis of (4-{[(β-D-galactopyranosyl)oxy]methyl}furan-3-yl)methyl hydrogen sulfate (GSF), which is a bifunctional saccharide mimetic consisting of a bis-hydroxymethylated furan core, a galactose residue and a sulfate group. It represents a mimetic of the GAG-subunit and may interact with the cell-surface or the ECM. We report the inhibitory capacity of the lead compound GSF to block adhesion and migration both of tumor cells and vascular endothelial cells and endothelial-cell-mediated angiogenesis. Surprisingly, GSF may not only block carbohydrate–protein interactions but also integrin-mediated protein–protein interactions, and thus, represents a strong candidate for the design of saccharide mimetics to be used as anti-tumor drugs.

## Results and Discussion

### Syntheses of saccharide mimetics

Glycosylation of 3,4-bis(hydroxymethyl)furan (**1**) with 1.0 equiv imidate **2** in CH_2_Cl_2_ afforded 48% of monosaccharide **3**. The synthesis of (4-{[(β-D-galactopyranosyl)oxy]methyl}furan-3-yl)methyl hydrogen sulfate (GSF, **5**) was modified by using trimethylamine sulfur trioxide instead of pyridinsulfoxide. The advantage was a short reaction time of 5 h at 50–55 °C instead of at room temperature (rt). One equiv of the benzoylated furan galactoside **3** was reacted with five equiv of trimethylamine sulfur trioxide in DMF at 55 °C. After purification on silica with CH_2_Cl_2_/CH_3_OH (5:1) the sulfate **4** was isolated in a yield of 95%.

Deprotection of the sulfated benzoylated furan **4** was carried out with freshly prepared sodium methylate in methanol at rt. After 10 h GSF (**5**) was obtained in a yield of 85%. It was found to be important that after the debenzoylation the aqueous medium be neutralized with 0.1 M HCl under control of a pH-meter, to pH 7.2. Under the highly acidic conditions induced by a Dowex H^+^ ion-exchange resin, the free sulfate decomposes. The product was purified by silica gel chromatography (CH_3_CN/H_2_O, 95:5).

**Scheme 1:**
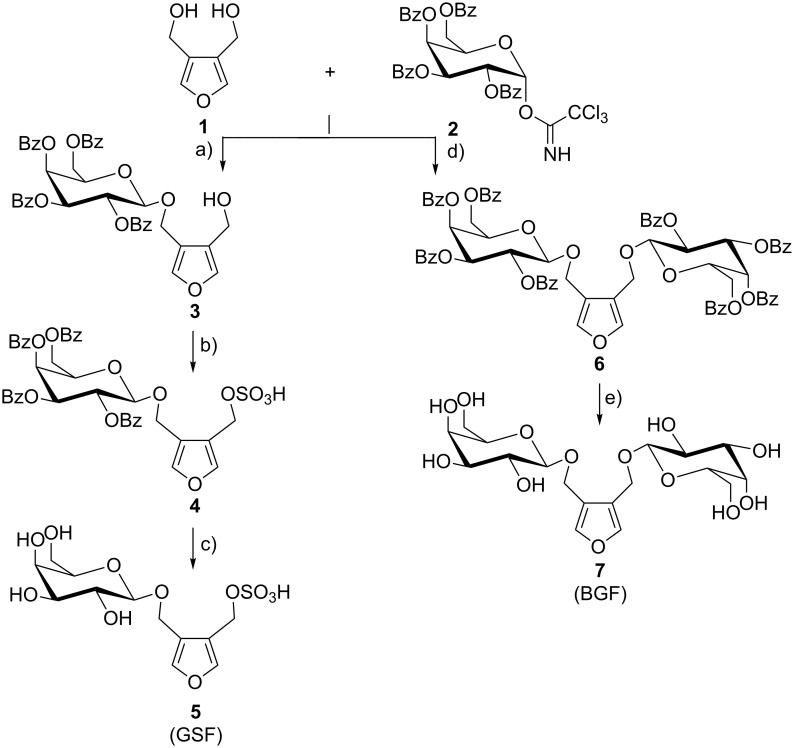
Synthesis of (4-{[(β-D-galactopyranosyl)oxy]methyl}furan-3-yl)methyl hydrogen sulfate (GSF, **5**) and 3,4-bis{[(β-D-galactopyranosyl)oxy]methyl}furan (BGF, **7**); (a) 1 equiv 3,4-bis(hydroxymethyl)furan (**1**) and 1 equiv imidate **2** in CH_2_Cl_2,_ TMSOTf, 0 °C, 2 h, yield: 48%; (b) 1 equiv **3** with 5 equiv NMe_3_·SO_3_ in DMF, 55 °C, 5 h, yield: 95%; (c) NaOMe in MeOH, 10 h, rt, adjustment to pH 7.2 with 0.1 M HCl, yield: 85%; (d) 1 equiv 3,4-(bis-hydroxymethyl)furan (**1**) and 2.5 equiv imidate **2** in CH_2_Cl_2,_ TMSOTf, 0 °C, 2 h, yield: 60%; (e) NaOMe in MeOH at 50 °C, neutralization with Dowex WX8 H^+^, yield: 61%.

3,4-bis{[(β-D-galactopyranosyl)oxy]methyl}furan (BGF, **7**) was synthesized by glycosylating **1** with 2.5 equiv of imidate **2** to yield 60% benzoylated BGF **6**. After deprotection with CH_3_ONa in CH_3_OH, the reaction mixture was neutralized by the addition of ion-exchange resin (Dowex WX8 H^+^) to yield 61% BGF (**7**) after recrystallization (Scheme 1).

### Analysis of cytotoxic effects of GSF toward human cells

**Figure 1:**
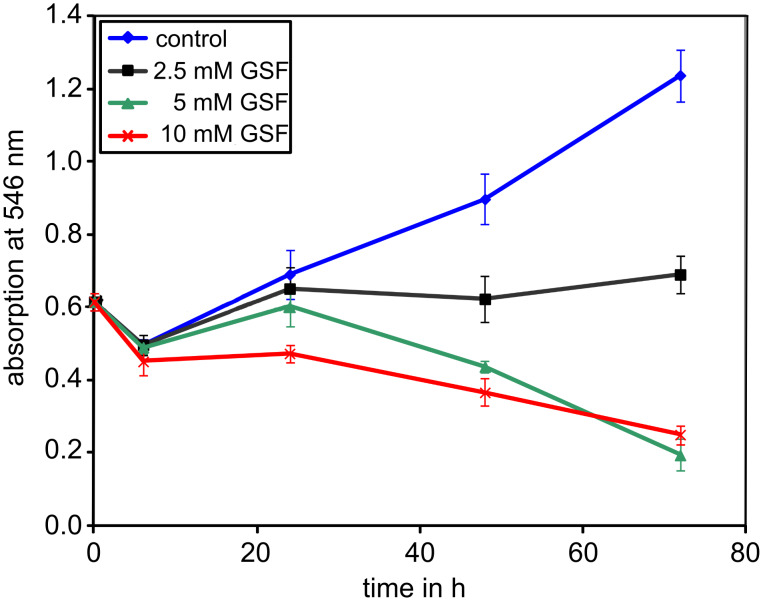
Effects of increasing concentrations of (4-{[(β-D-galactopyranosyl)oxy]methyl}furan-3-yl)methyl hydrogen sulfate (GSF) upon the growth of human melanoma cells WM-115. Cell densities were measured by staining adherent and fixed cells with sulforhodamine-B and solubilizing the dye with Tris base. Absorbance was determined at 546 nm. Absorbance values are proportional to cell numbers.

Two human melanoma cell lines, WM-115, isolated from a primary cancer, and WM-266-4, isolated from its cutaneous metastasis, were used to initially screen the compounds. To exclude the idea that the observed effects of the synthesized compounds on the cell properties were caused by the compound killing the cells, the cytotoxicity was determined over time with the sulforhodamine-B assay. As an example, the growth of WM-115 cells over a period of 72 h with increasing concentrations of GSF is shown in Figure 1.

GSF had a cytostatic effect at 2.5 mM beyond 24 h and exhibited cytotoxic effects after 48 h at 5 mM. We also performed cytotoxicity assays by counting cells after trypan-blue staining for disrupted membranes, but the standard deviations are quite high with this assay. Here, 5 mM GSF resulted in 40% dead WM-266-4 cells after 48 h, while only 10% of the WM-115 cells were dead. At earlier time points, which are relevant for the assays described below, only 10 mM GSF was toxic to WM-266-4, and lower concentrations had no effect. The melanoma cells isolated from the cutaneous metastasis therefore seem to be a bit more sensitive. Therefore, GSF is a mildly toxic compound with an IC_50_ of more than 10 mM after 48 h.

In the cell adhesion assays described, cells were exposed to GSF for a very short period of 2 h. Up to 6 h no effect of GSF up to a concentration of 10 mM was observed (Figure 1). In addition, the viability of the cells in cell adhesion assays after incubation with the test compounds was checked in each assay by using trypan blue, and even at the highest concentration of 40 mM no cytotoxicity was detectable.

BGF up to a concentration of 40 mM was neither cytotoxic nor cytostatic to cells of these two melanoma lines. The human endothelial cells HBMEC-60 showed a similar sensitivity to GSF as the WM-115 cells, for which incubations beyond 72 h led to the arrest of cell growth at 5 mM, and 10 mM was cytotoxic after these long incubation times.

### Inhibition of melanoma cell adhesion by a sulfated saccharide mimetic

In order to investigate the potential of the synthesized saccharide mimetics to interfere with the binding of the human melanoma line WM-115 to ECM proteins, we used a standard adhesion assay. Cells were radioactively labeled with [methyl-^3^H]-thymidine and we found a linear relation between the incorporated radioactivity and the number of cells, between 1 × 10^4^ and 7 × 10^4^ cells. To find the optimal conditions for WM-115 cells, their adhesion to increasing amounts of fibrinogen and fibronectin from 0.1–2 µg per well was measured. WM-115 cells showed maximum adhesion to both proteins at amounts of 0.5 µg/well, when wells were coated as described in the experimental section. The number of adherent cells varied between 40% and 70% of the 5 × 10^4^ cells added to one well. Reproducible values of around 50% adherence of cells were reached when cells were grown for 48 h before radioactive labeling. In general, more cells adhered to fibronectin than to fibrinogen.

WM-266-4 cells derived from the metastasis show a completely different growth and adhesion behavior compared to the cells isolated from the primary melanoma. Only 20–30% of the cells seeded on fibronectin or fibrinogen attached after 1 h. Results with inhibitors were therefore very difficult to perform, because the variation in attached cells in control wells was very high. Under normal culture conditions we always observed a lot of detached but viable cells. In wound-healing assays WM-266-4 cells very rapidly closed the wounds but were never as dense as WM-115 cells upon confluence. We therefore only used WM-115 cells for adhesion assays. These properties may point to high motility of a metastasis-derived cell line.

**Figure 2:**
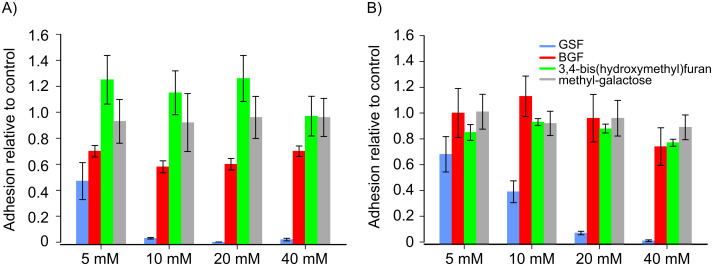
Inhibition of adhesion of WM-115 cells to fibrinogen (A), or to fibronectin (B) with increasing concentrations of (4-{[(β-D-galactopyranosyl)oxy]methyl}furan-3-yl)methyl hydrogen sulfate (GSF) (blue bars), 3,4-bis{[(β-D-galactopyranosyl)oxy]methyl}furan (BGF) (red bars), 3,4-bis(hydroxymethyl)furan (green bars), or methyl β-D-galactopyranoside (grey bars). Radioactively labeled cells were exposed to the compounds in medium for 1 h, then aliquots of the medium with 5 × 10^4^ cells were seeded into a well precoated with the extracellular matrix protein and incubated for a further 1 h. Nonadherent cells were washed off and adherent cells quantified by liquid scintillation counting. Means of four values ± SD relative to cells adhering in the absence of the compounds are shown.

Our earlier results had shown oligosaccharide mimetics containing furan as the core molecules to modulate cell–ECM interactions. Especially 3,4-bis{[(β-D-galactopyranosyl)oxy]methyl}furan (BGF) had shown bioactivity by blocking the adhesion of murine B16F10 melanoma cells to murine fibronectin [15]. We have now obtained similar results with the human WM-115 melanoma, for which we observed a 30% reduction of the adhesion to human fibrinogen without any dose dependence between 5–40 mM BGF (Figure 2A). One-way ANOVA analysis of variance showed the inhibition to be significant (p < 0.001) pointing to an optimal concentration of 10–20 mM BGF. In the presence of GSF we obtained a significant concentration-dependent decrease of adhesion to fibrinogen (one-way ANOVA p < 0.001), down to 3% adherent cells with 10 mM GSF. No inhibition could be seen with methyl β-D-galactopyranoside or 3,4-bis(hydroxymethyl)furan, demonstrating the importance of the sulfate- and the galactosyl-group as bioactive parts of the molecule.

The cell adhesion to fibronectin decreased significantly (one-way ANOVA p < 0.001) in a dose-dependent manner in the presence of GSF until cell binding was completely blocked at 40 mM (Figure 2B). No significant inhibition by either BGF, 3,4-bis(hydroxymethyl)furan, or methyl β-D-galactopyranoside was observed. In both experiments data analysis by two-way ANOVA showed significance for substance and concentration both with p < 0.001. The extent of inhibition of adhesion to fibronectin by GSF was weaker than to fibrinogen.

In the GSF molecule both the sugar and the sulfate moiety can interfere in several ways with cell–ECM binding. Carbohydrate–protein interactions may be inhibited by GSF binding to cell surface lectins, thereby perturbing the binding of the cell to the highly glycosylated ECM proteins [20], or by binding to lectin-like domains on fibronectin. The negatively charged sulfate group could also block cell–ECM interactions by interfering with heparin binding sites on fibronectin [21] and fibrinogen [22], which recognize the sulfated groups of cell-surface-expressed GAGs. GSF could mimic the highly sulfated GAG-building block. The result would be a reduction of free binding sites on the cell surface for natural ligands, such as sialyl-Lewis^x^, sialyl-Lewis^a^ or GAG.

By completely blocking the cell adhesion of WM-115 cells to fibrinogen and fibronectin with GSF we inhibited all interactions of cells with the ECM. To characterize the affected interactions in more detail, cells were incubated with peptidic integrin ligands and GSF in combination.

The RGD motif (an arginine–glycine–aspartic acid peptide sequence) is recognized and bound by several integrins including α_v_β_3_ and α_5_β_1_. The sequence glutamic acid–isoleucine–leucine–aspartic acid–valine (EILDV) is the binding motif of ligands of the α_4_β_1_ integrin. Both motifs are part of the amino acid sequence of fibronectin.

In initial experiments we investigated the inhibition of cell adhesion to fibronectin with increasing concentrations of the RGD-containing peptide: glycine–arginine–glycine–aspartic acid–serine–proline (GRGDSP) or EILDV. Neither peptide, up to 2 mM, completely inhibited adhesion of WM-115 cells to fibronectin. We then decided to combine the peptidic integrin ligands with GSF to see if an antagonism or additive effect upon cell adhesion occurs. For these experiments we chose 1 mM GRGDSP, 2 mM EILDV and 5 mM GSF.

**Figure 3:**
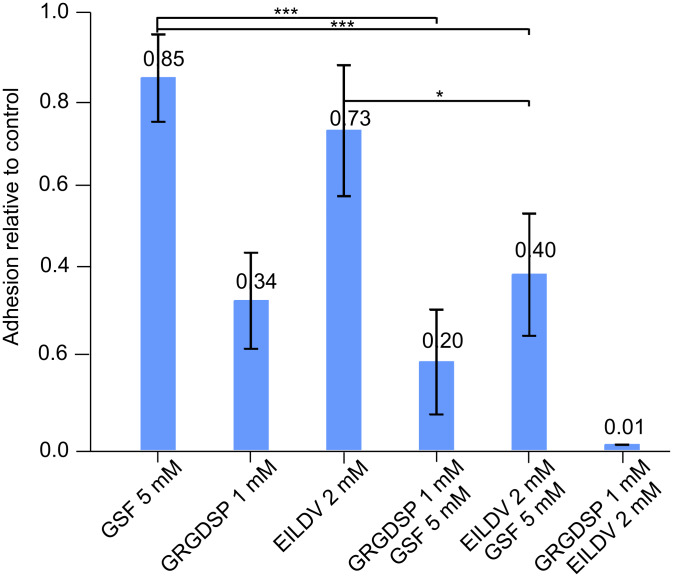
Inhibition of adhesion of melanoma cells WM-115 to fibronectin-coated plastic by 5 mM (4-{[(β-D-galactopyranosyl)oxy]methyl}furan-3-yl)methyl hydrogen sulfate (GSF), 1 mM glycine–arginine–glycine–aspartic acid–serine–proline (GRGDSP), 2 mM glutamic acid–isoleucine–leucine–aspartic acid–valine (EILDV), 1 mM GRGDSP plus 5 mM GSF, 2 mM EILDV plus 5 mM GSF, 1 mM GRGDSP plus 2 mM EILDV. Cells were radioactively labeled and incubated in the presence of the compounds indicated for a total of 2 h. Nonattached cells were then washed away and the remaining cells quantified by their radioactivity. Means of four values ± SD relative to cells adhering in the absence of the compounds are shown. One-way ANOVA followed by Bonferroni’s Multiple Comparison Test showed combinations with GSF to be significant as indicated; * = p < 0.05, *** = p < 0.001.

A complete inhibition of cell adhesion to fibronectin was observed in the presence of 1 mM GRGDSP plus 2 mM EILDV (Figure 3), and, as shown in Figure 2B, with GSF concentrations of 40 mM. On the one hand, there was no detectable carbohydrate–protein interaction, because the combined synthetic peptidic integrin ligands completely blocked cell adhesion to fibronectin, but on the other hand, cell adhesion could be inhibited with the sulfated saccharide mimetic GSF, probably by interfering with protein–protein interactions. The effects of the single agents were additive, showing that they probably interact at different sites that are important for adhesion. The effect of GSF on cell adhesion is dependent on ECM proteins, since WM-115 cell adhesion on plastic was not influenced by GSF up to 20 mM. At 40 mM, only 40% inhibition of adhesion was observed (data not shown).

Moitessier et al. [23] synthesized a combinatorial library of carbohydrate mimetics, based on xylose, as inhibitors of the integrins α_IIb_β_3_ and α_v_β_3_ and blocked binding to the natural ligand RGD, as reviewed by Gruner et al. [24]. Gottschalk and Kessler [25] created a β-D-mannose-containing inhibitor of α_4_β_1_ based on in silico modeled structures_._

### In silico analysis of GSF interaction with integrins

Our data showing similar inhibition of melanoma cell attachment to fibronectin by GSF as by the combined integrin ligand peptides, also points to an interaction of GSF with integrins. To find possible binding sites for GSF, we used a “blind docking” approach to screen the protein surface of the extracellular domain of α_v_β_3_, the crystal structure of which was published by Xiong et al. [26]. In an initial validation study, we performed multiple blind-docking experiments using the cyclic peptide Cilengitide^®^ (cyclo-[RGDfN(Me)V]) [27] as a ligand. The orientation of the pentapeptide Cilengitide and its binding to the binding site formed by the α and β chains of α_v_β_3_ reproduced the published X-ray structure with high accuracy.

**Figure 4:**
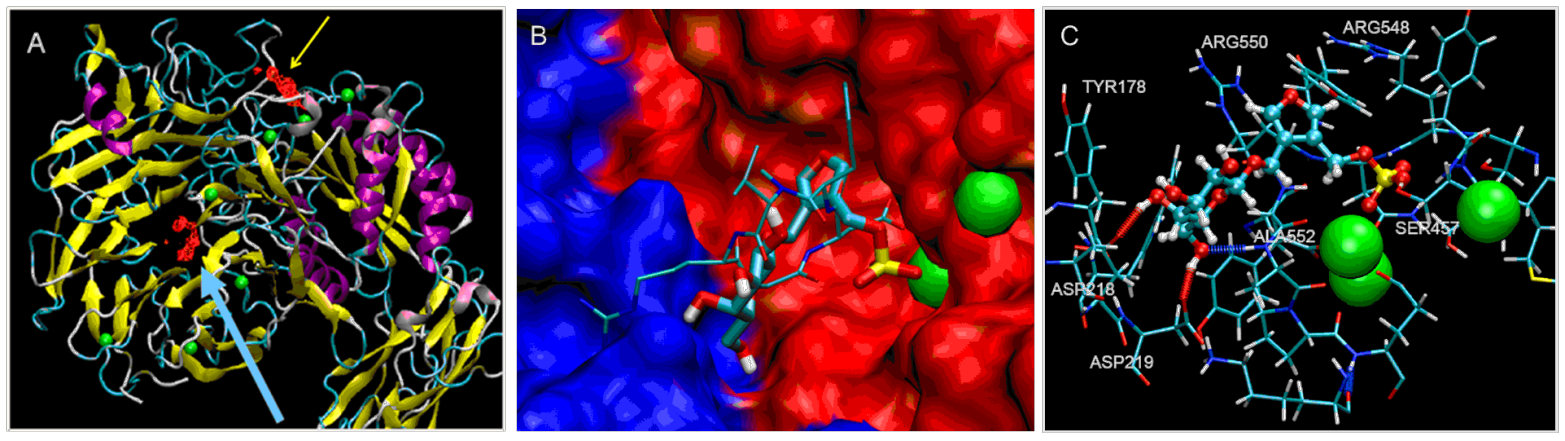
In silico blind-docking (A, B) and molecular dynamic simulations (C) of (4-{[(β-D-galactopyranosyl)oxy]methyl}furan-3-yl)methyl hydrogen sulfate (GSF) on the extracellular domain of the integrin α_v_β_3_. A: The blind-docking studies showed a preferred binding of GSF (red) to the binding site of the natural peptide ligands on α_v_β_3_ (yellow arrow). An additional binding site was predicted to be located inside the β-propeller domain of the α chain (blue arrow). B: Close-up of the binding site between the α_v_ (blue) and the β_3_ (red) chain of the integrin showing the synthetic pentapeptide cyclo-[RGDfN(Me)V] with thin lines and GSF in broader lines. Carbon, oxygen, nitrogen and sulfur are shown in blue, red, dark blue, and yellow, respectively. The manganese ions are represented as green spheres. C: Molecular dynamics simulation in water of the GSF molecule showing hydrogen bonds of the galactose moiety with the amino acids in the binding site of integrin α_v_β_3_ (Gal(OH4) with ASP218 and Gal(OH6) with ASP219/ALA552). The sulfate coordinates with one of the manganese ions.

The method thus validated was then used to dock GSF in silico to the same surface area. This resulted in two binding sites. One site was identical to the binding site described for the RGD motif [28] of the cyclic peptide and a second binding site was located inside the β-propeller domain of the α_v_ domain (Figure 4A). This binding site is probably a nonfunctional site since it is most likely not accessible for “surface bound” molecules such as ECM proteins. It is therefore possible that GSF blocks the functional binding site of natural ligands and the synthetic cyclic peptide Cilengitide (Figure 4B), explaining the observed interference with the adhesion of melanoma (Figure 2) and endothelial cells (Figure 7) to the extracellular matrix protein.

In order to investigate the molecular interactions of GSF in the RGD binding site of the integrin molecule in more detail we performed molecular dynamics (MD) simulation in explicit water. The MD simulation was started from the docked position of the GSF ligand. GSF slightly changed its position during the MD simulation, in which the sulfate group moved to the position that was occupied by the carboxylate group of the aspartic acid in the cyclic peptide (Figure 4C). The galactose forms several hydrogen bonds with the protein. A surface-water density analysis based on a MD simulation of the unligated protein in water revealed that the furan oxygen atom in GSF occupies the position of a (predicted) surface water-binding site.

### Impact of (4-{[(β-D-galactopyranosyl)oxy]methyl}furan-3-yl)methyl hydrogen sulfate (GSF) on melanoma cell migration

Migration of malignant tumor cells is an important step in the process of metastasis. Diverse members of the integrin family as well as MMPs have been shown to play a crucial role in the motility of cells [29–31]. Using a two-dimensional migration assay, we observed the effect of GSF on migration of the human melanoma lines WM-115 and WM-266-4 into a wound scratched into an intact cell monolayer.

**Figure 5:**
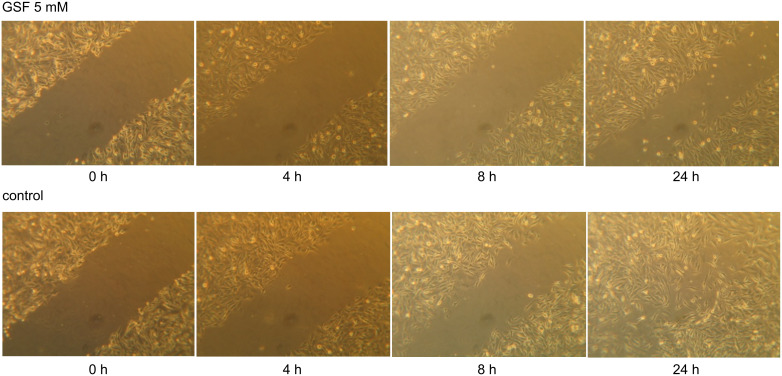
Intact cell monolayers of WM-115 cells in 12-well plates were wounded with a 100 µL pipette tip and washed three times with serum-free medium. Complete medium either containing 5 mM (4-{[(β-D-galactopyranosyl)oxy]methyl}furan-3-yl)methyl hydrogen sulfate (GSF) (upper row) or without GSF (lower row) was added to the wells, and cell migration into the wound was observed by microscopy after 0, 4, 8 and 24 h.

The migration rates of the two untreated cell lines are clearly different. While WM-115 cells from the original tumor needed about 24 h for the wound to close (Figure 5), the WM-266-4 cells derived from the metastasis of the same primary tumor are about three times faster, almost completely closing the wound within 8 h.

Incubation of the WM cells with GSF at concentrations that inhibit integrin-mediated cell adhesion, leads also to interference of the cell migration. GSF at a concentration of 5 mM diminished the number of WM-115 cells migrating into the wound after 24 h (Figure 5), and as shown in Figure 1 this is not due to a toxic effect of GSF. The influence on WM-266-4 cells was even more pronounced. These cells changed their morphology after 8 h by rounding up without complete detachment of the cells. After 24 h no more attached cells could be detected, but the detached cells were still viable (trypan-blue exclusion test) (data not shown). Binding of GSF to α_v_β_3_ probably leads to the loosening of focal adhesion plaques and detachment of cells from their adjacent matrix. This interaction would also explain a weaker effect of GSF on WM-115 cells, because their star-like, lamellipodic structure contains more focal adhesions than the filopodia of WM-266-4. These results confirm the assumption of an antagonistic effect of GSF on cell-surface molecules such as integrins.

### Impairment of MMP-2 activity by GSF

Matrix metalloproteinases (MMP) play a critical role in the invasion of tumor cells and metastasis formation. MMP-2, an ECM degrading gelatinase, is directly associated with tumor progression in a variety of cancer diseases [32]. The importance of active MMP-2 in melanoma progression is summarized in a review [33].

**Figure 6:**
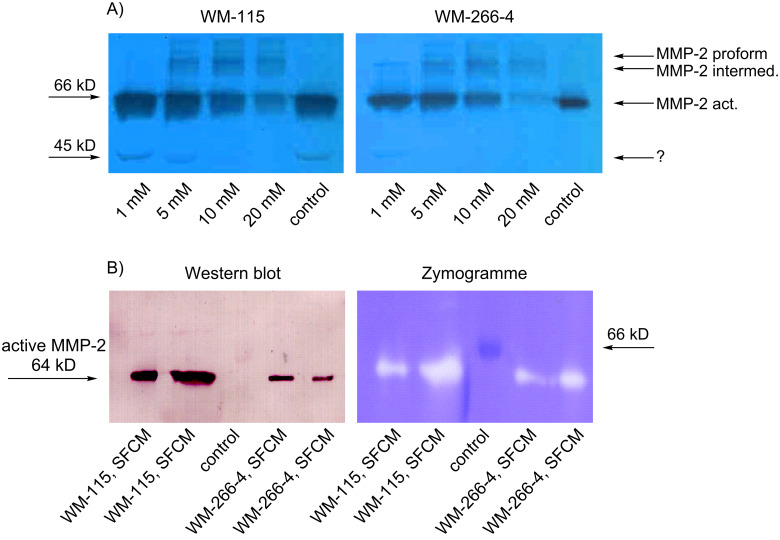
A: Zymograms (color inverted) of serum-free conditioned medium of melanoma cells treated with (4-{[(β-D-galactopyranosyl)oxy]methyl}furan-3-yl)methyl hydrogen sulfate (GSF) for 24 h at the concentrations indicated. Dark bands are the result of gelatinolytic activity of the proteins migrating into the gel. Molecular weights in kilodalton (kD) are indicated on the left. B: Western blot on the left analyzed with anti-MMP-2 antibodies showing that the gelatinolytic activity determined in parallel in the gel on the right is caused by activated MMP-2. “Control” lane is a molecular weight standard (albumin).

Because GSF inhibited the α_v_β_3_-mediated adhesion of WM-115 cells to fibrinogen, it may also influence the activation of MMP-2. The enzymatic activity of gelatinases such as MMP-2 can be determined in serum-free medium in which cells were grown for several days, i.e., serum-free conditioned medium (SFCM). After separation of proteins by electrophoresis in a polyacrylamide gel containing gelatin, the gelatinolytic activity can be determined after renaturation of the enzymes and visualized by staining the gel with Coomassie blue, in which white bands in a blue gel show enzymatic activity at the molecular weight of the enzymes. This process is called zymography and was performed to study the activation status of MMP-2 after incubation of the melanoma cells with GSF. Functionally active MMP-2 (64 kD) was found in SFCM in which WM-115 and WM-266-4 cells had been incubated while no bands of the proforms could be observed (Figure 6A, lanes „control“).

Bands were identified as MMP-2 in a Western blot with an anti-MMP-2 antibody (Figure 6B). With increasing concentrations of GSF the active MMP-2 form disappeared and the intermediate (68 kD) and inactive proform (72 kD) were detectable in SFCM from both cell lines (Figure 6A). In SFCM of WM-115 cells an unknown gelatinolytic band of 45 kD appeared in the samples, which disappeared also with higher concentrations of GSF. Incubation of the cells with 20 mM GSF showed an overall reduction of the gelatinolytic activity, probably due to a cytotoxic effect of GSF at higher concentrations in serum-free medium, which is a stressful condition for cells routinely cultivated in the presence of 10% fetal bovine serum (FBS) (Figure 1).

Brooks et al. [5] proposed an activation model for MMP-2 in which the gelatinase is recruited to the cell surface by the binding of pro-MMP-2 and active MMP-2 to integrin α_v_β_3_. After cleavage, active MMP-2 is liberated and degrades ECM components such as collagen type IV. Hofmann et al. [34] proposed a second activation model, which is also associated with binding of the pro-MMP-2 to α_v_β_3_ in addition to membrane-bound matrix metalloprotease MT1-MMP, suggesting that this integrin plays a crucial role in MMP-2 activation.

Teti et al. [35] observed increasing MMP-2 activity after incubation with the α_v_β_3_-integrin-ligand GRGDSP, similar to the activation seen upon binding of cells to fibronectin or fibrinogen. However, GSF, which inhibits cell adhesion to both ECM-proteins, inhibits the cleavage of the MMP-2 pro-sequence.

A GSF–α_v_β_3_ interaction could directly or allosterically inhibit binding of pro-MMP-2 to the integrin, and our in silico blind-docking studies (Figure 4) have shown GSF to bind to another domain on α_v_β_3_ in addition to the RGD binding site. Alternatively, GSF could bind to the ECM and thereby inhibit cell adhesion and MMP-2 activation. The lack of inhibition of cell adhesion to plastic by GSF points to such a mechanism. A reduction of the gelatinase MMP-2 and its proforms could also be caused by lower pro-MMP-2 expression upon GSF binding or its cellular uptake.

### Effects of saccharide mimetics on adhesion, migration and tubule formation of vascular endothelial cells

Endothelial cells play a central role in the process of angiogenesis, which is stimulated and regulated by a complex network of chemo- and cytokine-signaling. One of the pivotal angiogenic factors is the vascular endothelial growth factor (VEGF); in addition, the initial steps of angiogenesis may also be stimulated by inflammatory growth factors, such as the tumor necrosis factor (TNF) [35–39]. During the first stages of vessel formation endothelial cells have to adhere to and migrate along the extracellular matrix, build contacts between each other, and finally form tubules [40–41].

In order to evaluate the influence of saccharide mimetics on endothelial vessel formation we studied the effects of GSF and BGF in several in vitro endothelial cell assays. For our studies we used a transformed human endothelial cell line (HBMEC-60) derived from bone marrow [42], which, in its biological characteristics, is similar to primary endothelial cells, such as human umbilical cord vein endothelial cells (HUVEC) [19,43].

**Figure 7:**
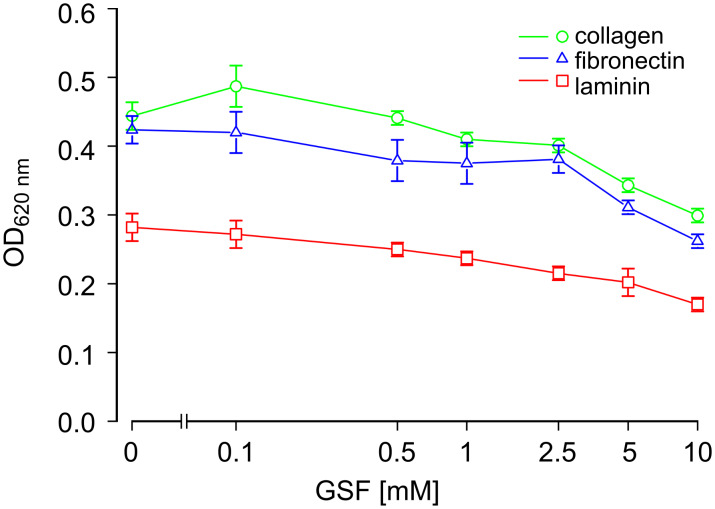
Adhesion of HBMEC-60 to extracellular matrix proteins. Prior to the adhesion experiments, HBMEC-60 cells were incubated for 30 min with (4-{[(β-D-galactopyranosyl)oxy]methyl}furan-3-yl)methyl hydrogen sulfate (GSF) at the concentrations indicated. The cells were then seeded onto plates coated with fibronectin, collagen or laminin. After 30 min nonadherent cells were removed, and adherent cells were fixed, stained with methylene blue, and washed; the cell-bound dye was then extracted and the absorption determined photometrically. In some experiments cells were also stimulated with TNF as described in the Experimental section. TNF pretreatment of cells resulted in virtually the same adhesive properties as without TNF and with a significant inhibition of adhesion by GSF at 5 mM. The values shown are the mean and SD of six replicates. The same results were obtained in two independent experiments.

The influence of GSF on the adhesion of HBMEC-60 endothelial cells to ECM components was tested using plates coated with fibronectin, laminin or collagen. Addition of GSF to the cells in medium inhibited the adhesion of these cells to all three matrix components in a dose-dependent fashion (Figure 7). Cell adhesion was strongest to fibronectin and collagen, and weaker to laminin in the absence of GSF. A significant inhibition of adhesion to fibronectin was observed at a concentration of 5 mM GSF (approximately 28% inhibition). This result is similar to that obtained with GSF-mediated inhibition of adhesion of WM-115 melanoma cells to fibronectin (Figure 2). At concentrations of 10 mM GSF adhesion to all three ECM components was inhibited by about 40%.

**Figure 8:**
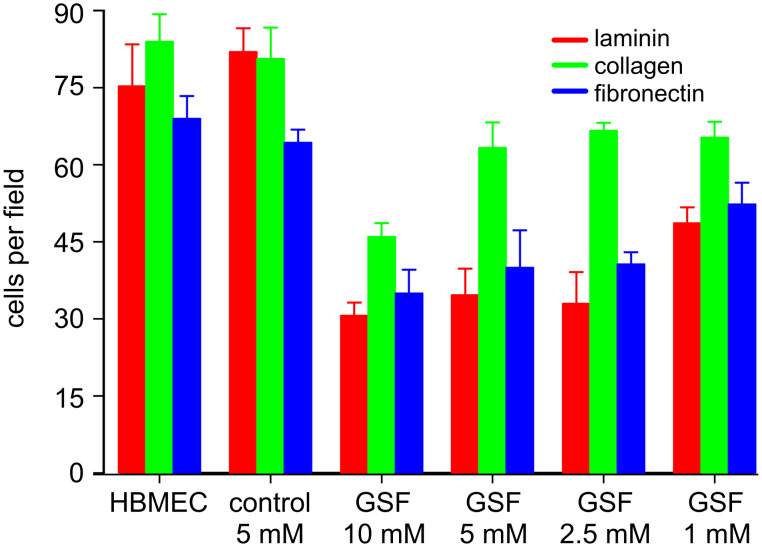
Effect of (4-{[(β-D-galactopyranosyl)oxy]methyl}furan-3-yl)methyl hydrogen sulfate (GSF) on transmigration of HBMEC-60 cells on transwell plates with 8 μm pores and adhesion to matrix components on the lower side of the wells. Cells were stimulated with TNF, then GSF or 3,4-bis(hydroxymethyl)furan (control), or medium only (HBMEC), were added. Cells were seeded into transwell inserts coated with extracellular matrix proteins as indicated. After incubation for 30 min nonadherent cells were washed away, and cells sticking to the proteins were fixed, stained and quantified. Shown is an exemplary experiment with TNF-stimulated HBMEC-60 cells. The experiments were performed in triplicate and in three independent experiments for each extracellular matrix protein.

In a subsequent step, we assessed the influence of GSF on the migration of HBMEC-60 cells through 8 μm pores in polycarbonate transwells coated with extracellular matrix proteins. For this assay endothelial cells were stimulated before seeding into the plates, with either VEGF or TNF as indicated. Inhibition of migration and adhesion to the lower side of the transwells was observed at a concentration of 1 mM GSF and increased up to 10 mM for all three matrix components, although to different extents. Two-way ANOVA analysis showed significant effects of concentration (p < 0.001) and substance (p < 0.001) on migration. One-way ANOVA showed significant (p < 0.001) concentration dependence for GSF on all three matrix proteins. The strongest inhibitory effects were obtained after TNF stimulation at a concentration of 10 mM GSF on collagen-coated plates (Figure 8). It has to be noted that BGF, though much weaker than GSF, also inhibited migration and adhesion (data not shown).

As described earlier, migration of WM-115 (Figure 5) and WM-266-4 melanoma cells observed in a two-dimensional migration assay system was also inhibited by GSF. It therefore seems that adhesion and migration both of human tumor and endothelial cells are affected by GSF, pointing to a more general mechanism, possibly mediated by several different forms of integrin that adhere to either, collagen, fibronectin or laminin. Besides perturbing integrin-ECM protein–protein interactions, GSF may also interfere with ionic forces between charged ECM proteins and cells due to the negative charge of the sulfate.

**Figure 9:**
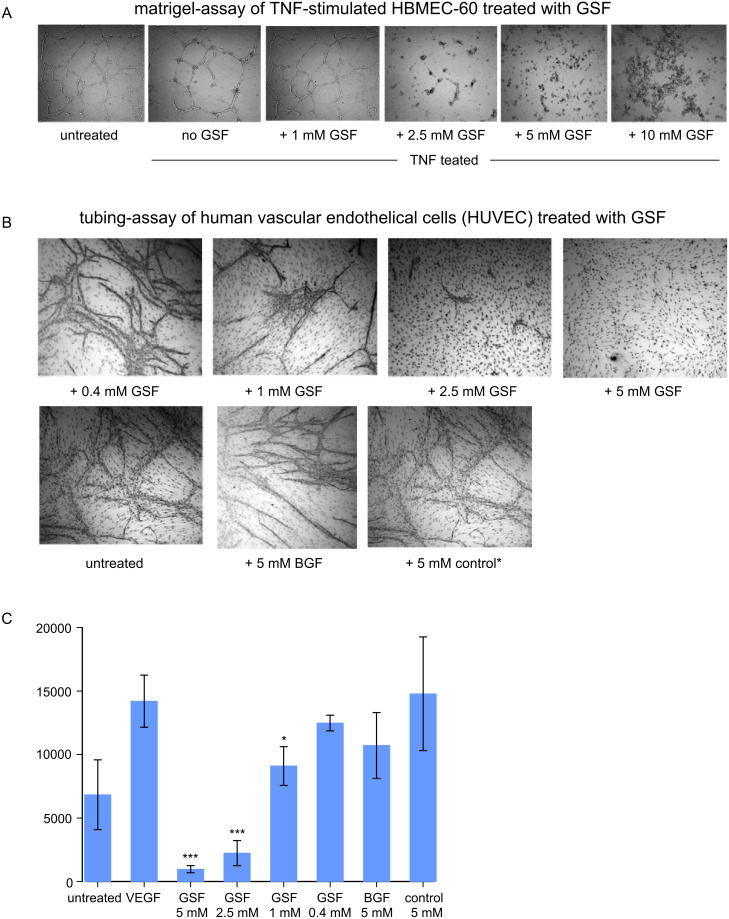
Influence of saccharide mimetics on endothelial networking (matrigel-assay) (A) and tube formation (B,C). A: The matrigel-assay is used to assess the initial stages of tube formation, i.e., the lining-up and subsequent formation of networks between endothelial cells. In the experiment shown, cells were stimulated with TNF or left untreated for 24 h, then (4-{[(β-D-galactopyranosyl)oxy]methyl}furan-3-yl)methyl hydrogen sulfate (GSF) was added in the concentrations indicated. The cells were then seeded onto the matrigel, which consists of a solubilized complex basement membrane of the EHS mouse tumor [44], and incubated overnight. B: The tubing assay measures the formation of endothelial tubules in a coculture of HUVEC and fibroblast cells (dots seen in the background). VEGF, which is necessary for proper in vitro tubule formation, was added at day 0 to the cells together with GSF or 3,4-bis{[(β-D-galactopyranosyl)oxy]methyl}furan (BGF) in the concentrations indicated. As one control, tubule formation is shown with VEGF only (untreated) or with 3,4-bis(hydroxymethyl)furan (5 mM control*). The growth media including growth factors and the respective mimetics were changed at days 4 and 7. At day 9 the medium was removed and the endothelial cells were stained with a monoclonal antibody against the cell surface antigen CD31 to allow evaluation of tubule formation. The assay was performed in three independent experiments with the same results in terms of the GSF effect. C: The tubule formation as observed under the microscopic was quantitatively analyzed using Angiosys 1.0 software, which counts the total tubule length, number of tubules and junctions between different tubules. Here, we show the results for total tubule length of an experiment performed as shown in panel B. The values are means and standard deviations of three experiments, * = p < 0.05, *** = p < 0.001 of the GSF exposed cells compared to VEGF alone. ANOVA one-way analysis of variance showed the concentration dependence of the GSF effect to be significant with p < 0.0001. The results for the other parameters reflected the same tendency of inhibitory capacity of GSF with increasing concentrations.

In order to investigate cell biological features that are more specific for endothelial cells, we looked at contact and network formation between endothelial cells under the influence of saccharide mimetics as measured in the matrigel-assay. The cells were prestimulated with either VEGF or TNF for 24 h and then added to the plates coated with a complex extracellular matrix (matrigel). Network formation of cells in the presence of mimetics was observed after an overnight incubation period. As shown in Figure 9A, a concentration of 2.5 mM GSF already severely disturbed the formation of interendothelial cell contacts, and at higher GSF concentrations no contacts at all were built. It has to be noted that at a concentration of 5 mM GSF, and to a somewhat lesser extent also at 10 mM GSF, the endothelial cells were still viable such that GSF disturbs the initial phases of their networking rather than their viability and proliferation.

Although the in silico studies described earlier predict an interaction of GSF with the RGD binding site on integrins, and integrin α_v_β_3_ antagonists have been shown to block angiogenesis [45], we cannot rule out that this saccharide mimetic also competes with cell-surface-bound oligosaccharides interacting with the respective binding partners on neighboring cells. In this respect our results are reminiscent of a previous study in which we described the inhibition of endothelial networking by monoclonal antibodies against the histo blood group oligosaccarides Lewis^y^ and H and further by siRNA inhibition of the fucosyltransferase FUT1 and GDP-4-keto-6-deoxymannose-epimerase/reductase (FX-protein) [19]. The FX-protein is required for de novo synthesis of GDP-fucose from GDP-mannose [46], whereas FUT 1 catalyzes the α_2_ fucosylation of blood group H type 1 and Lewis^y^ oligosaccharides. The fucosylated oligosaccharides Lewis^y^ and H are strongly expressed on the cellular extensions that form the first contacts between endothelial cells. From these experiments we concluded that fucosylation of oligosaccharide chains is necessary for the first steps in the lining-up of endothelial cells and ensuing tubule formation. In our earlier study we reported that bis-glycosylated 3,4-bis(hydroxymethyl)furan derived saccharide mimetics containing fucose and galactose or two galactoses showed good structural agreements with the terminal oligosaccharides of Lewis^y^ by in silico MD simulation, and at the same time these mimetics showed biological activities [15]. Lewis^y^ has been reported to be part of the oligosaccharide moiety of the α_5_β_1_ integrin interacting with fibronectin [47]. Therefore, the inhibitory activity of GSF with regard to integrin-mediated processes may be caused by blockade of the RGD site as well as by interfering with the oligosaccharides of the respective integrin. Further studies are needed to show whether GSF indeed interacts with oligosaccharide binding sites on cells and therefore contributes to the inhibition of cell–cell communication.

As expected from the results of the matrigel assay, GSF also inhibited the sprouting of endothelial cells and their tubule formation in the long-term tube-formation assay of HUVEC cells cocultivated with fibroblasts in a dose-dependent manner (Figure 9B and Figure 9C). An inhibitory effect of GSF could be already observed at a concentration of 0.4 mM and increasingly in a dose-dependent manner up to 5 mM GSF. In contrast, even 5 mM BGF showed a very minor effect on tubule formation, indicating that GSF had a specific inhibitory effect on in vitro angiogenesis.

## Conclusion

We have described the synthesis of the lead compound (4-{[(β-D-galactopyranosyl)oxy]methyl}furan-3-yl)methyl hydrogen sulfate (GSF) and its possible biological application with regard to the inhibition of tumor cell adhesion to an extracellular matrix, and to interference with endothelial cell-mediated angiogenesis. GSF thus targets three important aspects of metastasis, namely tumor cell invasion, the adhesion of the cells to extracellular matrix proteins, and the ensuing angiogenesis enabling tumor growth. These properties make this lead compound interesting for further development as an anti-cancer drug. The integrins ανβ_3_ and α_5_β_1_ are highly expressed on activated endothelia of tumor tissues and, thus, represent ideal targets for cancer treatment. In this respect, GSF may occupy the same binding site on these integrins as the cyclic RGD pentapeptide Cilengitide^®^, which targets the integrins ανβ_3_ and α_5_β_1_. Cilengitide has been described as a strong inhibitor of angiogenesis and is currently under investigation in several clinical trials for the treatment of recurrent malignant glioblastoma [48]. GSF, due to its direct anti-tumor and anti-angiogenic effect, may represent a new class of small-molecule anticancer drugs. Although anti-angiogenic drugs such as the monoclonal antibody Avastin^®^, which blocks the activity of VEGF, are already in clinical use for tumor therapy, they cannot prevent overall progression of malignant cancers. This may be due to the tumors mounting a resistance against these treatments, e.g., by using mechanisms of blood-vessel formation other than VEGF-mediated ones. Therefore, there is a strong need to develop new anti-angiogenic drugs for tumor treatment. Saccharide mimetics, as shown here, may have an excellent potential, not only because of their inhibition of angiogenesis but also because of their ability to directly interfere with mechanisms that are essential for metastasis formation.

An advantage of saccharide mimetics such as GSF, also in comparison to more complex oligosaccharide mimetics, is their chemically easy accessibility and their potential for further derivatization. In this respect, GSF is a lead compound, which upon minor variations in its molecular structure, such as the introduction of a second sulfate or the replacement of the sulfate by a halogen, may become even more potent [49]. Further systematic tests are needed to investigate the structure–activity relationship.

## Experimental

### General

Commercially available reagents and anhydrous solvents were used without further purification unless stated otherwise. TMSOTf was from Acros, Geele, Belgium, all other chemicals were from either Sigma-Fluka, Taufkirchen, Germany, Fisher Scientific, Schwerte, Germany or Merck Schuchardt, Darmstadt, Germany. Thin-layer chromatography was performed on TLC plates Si 60 F_254_ (Merck) in petroleum ether (PE), ethyl acetate (EE) or other solvents as indicated, and compounds were visualized under UV and after spraying with a cerium-molybdate spray reagent (20 g ammonium molybdate, 0.4 g cerium(IV) sulfate in 400 mL 10% sulfuric acid) or vanillin–sulfuric acid spray reagent (1% vanillin in 15% sulfuric acid). Column chromatography was performed on silica gel (63–200 mesh, particle size 60 Å, MP Biomedicals, Eschwege, Germany), Dowex WX8 H^+^ was from Serva, Heidelberg, Germany. Analytical HPLC was performed on a Jasco HPLC system by using a Lichrosphere-RP18 (e)-5µ, 250/4 mm column for reversed-phase chromatography unless stated otherwise. The UV detector was set to 210 nm to monitor the signals of the analytes.

^1^H and ^13^C NMR spectra were recorded on a Bruker AM 250 (250 MHz for ^1^H and 63 MHz for ^13^C) instrument (Bruker, Rheinstetten, Germany) with Me_4_Si (δ = 0) as the internal standard. Mass spectrometry electrospray ionization (ESI–MS) measurements were recorded on a Finnigan MAT TSQ 7000 instrument. All measurements were performed at the central spectroscopy unit of the DKFZ.

**Synthesis of 3,4-bis{[(β-D-galactopyranosyl)oxy]methyl}furan (BGF, 7):** 2,3,4,6-Tetra-*O*-benzoyl-β-D-galactopyranose was synthesized by ChemCon GmbH (Freiburg, Germany). 2,3,4,6-Tetra-*O*-benzoyl-β-D-galactopyranosyl trichloroacetimidate (**2**) was synthesized according to Schmidt et al. [14]. 3,4-Bis(hydroxymethyl)furan (**1**) was synthesized according to [50].

For 3,4-bis{[(2,3,4,6-tetra-*O*-benzoyl-β-D-galactopyranosyl)oxy]methyl}furan (**6**) TMSOTf (100 µL) was added to a solution of furan **1** (1.28 g, 10 mmol) and imidate **2** (18.5 g, 25.0 mmol) in dry CH_2_Cl_2_ (150 mL) at −40 °C. The reaction was stirred for 2 h at 0 °C and then extracted with aq. NaHCO_3_ (100 mL) and H_2_O (100 mL). After evaporation of the solvent the product was recrystallized from PE/EE, 1:1. The bis-galactoside **6** (7.70 g, 6 mmol) was obtained in 60% yield as a white foam. TLC (PE/EE 3:1) *R*_f_ 0.1; ESI–MS (*m*/*z*): 1285.4 [M + H]^+^ (2), 1302.5 [M + NH_4_]^+^ (10), 1307.4 [M + Na]^+^ (100); HPLC: (*n*-hexane/THF 80:20→50:50 in 10 min, Purosphere-Si80-5µ, 125/4 mm) *t*_R_7.9 min; ^1^H NMR (250 MHz, CDCl_3_) δ 3.80 (dt, *J*_4´,5´_ = 1 Hz, *J*_5´,6a´_ = *J*_5´,6b´_= 6.5 Hz, 2H, 2 H5´), 4.11 (d, *J*_1´,2´_ = 7.8 Hz, 2H, 2 H1´), 4.21 (d, *J*_O-CHa,O-CHb_ = 11.4 Hz, 2H, 2 O-CH_a_), 4.34 (dd, *J*_6a´,6b´_ = 11.2 Hz, 2H, 2 H6_a_´), 4.57 (dd, 2H, 2 H6_b_´), 4.63 (d, 2H, 2 O-CH_b_), 5.41 (dd, 2H, *J*_2´,3´_ = 10.4 Hz, *J*_3´,4´_ = 3.5 Hz, 2 H3´), 5.66 (dd, 2H, 2 H2´), 5.84 (dd, 2H, 2 H4´), 8.09–7.19 (m, 42H, 40 Ar-H, H2, H5); ^13^C NMR (63 MHz, CDCl_3_) δ 61.62, 62.00 (2 C6´, 2 O-CH_2_), 68.06, 69.82, 71.15, 71.40 (2 C2´, 2 C3´, 2 C4´, 2 C5´), 99.68 (2 C1´), 120.32 (C3, C4), 128.30, 128.57, 128.71, 128.83, 129.00, 129.48, 129.63, 129.74, 129.87, 130.02, 133.29, 133.38, 133.59 (48 Ar-C), 142.41 (C2, C5), 164.90, 165.54, 165.98 (8 CO).

Deprotection to 3,4-bis{[(β-D-galactopyranosyl)oxy]methyl}furan (BGF, **7**): The bis-galactoside **6** (2.00 g, 1.56 mmol) was added to a freshly prepared solution of sodium (0.5 g) in methanol (50 mL). The resulting suspension was heated to 50 °C until it was clear. When debenzoylation of **6** was complete (monitored by TLC) the solution was neutralized with Dowex WX8 H^+^, the solvent evaporated, the product dissolved in H_2_O (50 mL) and extracted twice with diethylether (50 mL), the H_2_O evaporated and the product crystallized from methanol. BGF (**7**, 430 mg, 0.95 mmol) was obtained with 61% yield as a white foam. TLC (CHCl_3_/EtOH 4:1) *R*_f_ 0.8; ESI–MS (*m*/*z*): 453.0 [M + H]^+^ (12), 470.1 [M + NH_4_]^+^ (28), 475.0 [M + Na]^+^ (100), 922.5 [2M + H]^+^ (2), 927.4 [2M + Na]^+^ (5); HPLC: (CH_3_CN/H_2_O 0:100→100:0 in 40 min) *t*_R_ 5.8 min; ^1^H NMR (250 MHz, D_2_O) δ 3.53 (dd, *J*_1´,2´_ = 7.7 Hz, *J*_2´,3´_ = 9.9 Hz, 2H, 2 H2´), 3.63 (dd, *J*_3´,4´_ = 3.4 Hz, 2H, 2 H3´), 3.69 (ddd, *J*_4´,5´_ = 0.9 Hz, *J*_5´,6a´_ = 4.3 Hz, *J*_5´,6b´_ = 7.8 Hz, 2H, 2 H5´), 3.75 (dd, *J*_6a´,6b´_ = 11.5 Hz, 2H, 2 H6_a_´), 3.82 (dd, 2H, 2 H6_b_´), 3.92 (dd, 2H, 2 H4´), 4.47 (d, 2H, 2 H1´), 4.71 (d, *J*_O-CHa,O-CHb_ = 11.8 Hz, 2H, 2 O-CH_a_), 4.92 (d, 2H, 2 O-CH_b_), 7.64 (s, 2H, H2, H5); ^13^C NMR (63 MHz, D_2_O) δ 65.58, 65.80 (2 C6´, 2 O-CH_2_), 73.25, 75.26, 77.39, 79.73 (2 C2´, 2 C3´, 2 C4´, 2 C5´), 105.90 (2 C1´), 124.83 (C3, C4), 148.07 (C2, C5).

**Synthesis of (4-{[(β-D-galactopyranosyl)oxy]methyl}furan-3-yl)methyl hydrogen sulfate (GSF, 5):** 3-Hydroxymethyl-4-{[(2,3,4,6-tetra-O-benzoyl-β-D-galactopyranosyl-1-yl)-oxy]methyl}furan (**3**) was synthesized by adding TMSOTf (100 µL) to an ice cooled solution of furan **1** (2.92 g, 22.3 mmol) and imidate **2** (17.2 g, 22.3 mmol) in dry CH_2_Cl_2_ (150 mL). The reaction was stirred for 2 h at 0 °C, and 1 h at rt, and then stopped by neutralization with triethylamine. The solvent was evaporated and the product purified by silica gel column chromatography (PE/EE = 7:3→1:1). The monoglycoside **3** (7.2 g, 10.1 mmol) was obtained with 48% yield as a white foam. TLC (PE/EE 7:3) *R*_f_ 0.27; HPLC: (CH_3_CN/H_2_O, 0:100→100:0 in 60 min) *t*_R_ 53 min; ESI–MS (*m*/*z*): 729.4 [M + Na]^+^ (100); ^1^H NMR (250 MHz, CDCl_3_) δ 4.36 (m, 3H, CH_2_-furan, H5´), 4.46 (dd, *J*_5´,6a´_ = 6.3 Hz, *J*_6a´,6b´_ = 11.4 Hz, 1H, H6_a_´), 4.67 (d, *J* = 12.2 Hz, 1H, CH_a_OGal), 4.70 (dd, *J*_5´,6b´_ = 6.5 Hz, 1H, H6_b_´), 4.88 (d, 1H, CH_b_OGal), 4.94 (d, *J*_1´,2´_ = 7.9, 1H, H1´), 5.61 (dd, *J*_2´,3´_ = 10.4 Hz, *J*_3´,4´_ = 3.7 Hz, 1H, H3´), 5.84 (dd, 1H, H2´), 6.00 (dd, 1H, H4´), 7.4–8.12 (m, 22H, 20 Ar-H, H2, H5); ^13^C NMR (63 MHz, CDCl_3_) δ 55.62 (CH_2_OH), 62.21 (CH_2_OGal), 69.72, 71.15, 71.2, 76.38 (C2´, C3´, C4´, C5´), 99.7 (C1´), 120.26 (C3), 124.2 (C4), 128.1–133.6 (20 × CBz), 142.4 (C2, C5), 164.7–166.1 (4 × CO).

Synthesis of (4-{[2,3,4,6-tetra-O-benzoyl-β-D-galactopyranosyl)oxy]methyl}furan-3-yl)methyl hydrogen sulfate (**4**): To a solution of furan **3** (5.0 g, 7.08 mmol) in dry DMF (35 mL), NMe_3_·SO_3_ (4.92 g, 35.4 mmol) was added. The reaction mixture was stirred for 5 h at 55 °C, the solvent evaporated and the product purified by silica gel column chromatography (CHCl_3_/MeOH 5:1). Compound **4** (5.4 g, 6.8 mmol) was obtained in 95% yield as a white solid. TLC (CHCl_3_/MeOH 5:1) *R*_f_ 0.1; ESI–MS (*m*/*z*): 785.2 [M − H]^−^ (100); HPLC: (CH_3_CN/H_2_O 0:100→100:0 in 60 min) *t*_R_ 34 min; ^1^H NMR (250 MHz, CDCl_3_) δ 4.31 (dd, *J*_4´,5´_ = 1.4 Hz, *J*_5,6a´_ = *J*_5´,6b´_ = 6.9 Hz, 1H, H5´), 4.38 (dt, *J*_6a´,6b´_ = 12.2 Hz, 1H, H6a´), 4.48 (d, *J*_CHaOGal,ChbOGal_ = 12.8 Hz, 1H, CH_a_OGal), 4.63 (dd, 1H, H6b´), 4.65 (d, 1H, CH_b_OGal), 4.77 (s, 2H, CH_2_OSO_3_), 4.96 (d, *J*_1´,2´_ = 7.1 Hz, 1H, H1´), 5.64 (dd, *J*_2´,3´_ = 11.0 Hz, *J*_3´,4´_ = 2.8 Hz, 1H, H3´), 5.69 (dd, 1H, H2´), 5.93 (dd, 1H, H4´), 7.6–8.0 (m, 22H, 20 Ar-H, H2, H5); ^13^C NMR (63 MHz, CDCl_3_) δ 55.06 (CH_2_OSO_3_), 61.48 (C6´), 62.07 (CH_2_OGal), 68.11, 69.08, 70.08, 71.51 (C2´, C3´, C4´, C5´), 100.01 (C1´), 119.23, 120.78 (C3, C4), 128.1–133.5 (20 × CBz), 142.1, 143.06 (C2, C5), 165.50, 165.60, 165.68, 166.64 (4 × CO).

Deprotection to (4-{[(β-D-galactopyranosyl)oxy]methyl}furan-3-yl)methyl hydrogen sulfate (GSF, **5**): Solid, powdered NaOCH_3_ (170 mg, 3.15 mmol) was added to a solution of furan **4** (1.0 g, 1.27 mmol) in dry methanol (20 mL), and the reaction mixture was stirred for 10 h at rt. The solvent was then evaporated, and the product was dissolved in water (10 mL), adjusted to pH 7.2 with 0.1 M HCl, and extracted three times with diethyl ether. The aqueous layer was lyophilized. GSF (**5**, 400 mg 1.08 mmol) was obtained with 85% yield as a white powder. TLC (CH_3_CN/H_2_O 9:1): *R*_f_ 0.7; HPLC: (CH_3_CN/H_2_O 2:98) *t*_R_ 5 min; ESI–MS (*m*/*z*): 369.0 [M − H]^−^ (100); ^1^H NMR (250 MHz, D_2_O) δ 3.53 (dd, *J*_2´,3´_ = 9.8 Hz, *J*_1´,2´_ = 7.7 Hz, 1H, H2´), 3.64 (dd, *J*_3´,4´_ = 3.2 Hz, 1H, H3´), 3.73 (ddd, *J*_5´,6a´_ = 5.9 Hz, *J*_5´,6b´_ = 6.3 Hz, *J*_5´,4´_ = 1.6 Hz, 1H, H5´), 3.81 (dd, *J*_6a´,6b_ = 12.4 Hz, 1H, H6a´), 3.83 (dd, 1H, H6b´), 3.94 (dd, 1H, H4´), 4.48 (d, 1H, H1´), 4.68 (d, *J*_CHaOGal,ChbOGal_ = 11.5 Hz, 1H, CH_a_OGal), 4.90 (d, 1H, CH_b_OGal), 5.07 (s, 2H, CH_2_OSO_3_), 7.65 (s, 1H, H5), 7.68 (s, 1H, H2); ^13^C NMR (63 MHz, DMSO-*d*_6_) δ 58.4, 60.5, 60.6 (CH_2_OSO_3_, CH_2_OGal, C6´), 68.2, 70.6, 73.4, 75.2 (C2´, C3´, C4´, C5´), 102.6 (C1´), 121.3, 121.7 (C3, C4), 141.49, 141.52 (C2, C5).

### In silico blind-docking and molecular dynamics simulations

A flexible docking approach using AUTODOCK 3.05 [51] was applied to screen a large part of the surface of the protein for potential binding sites. The complete β-propeller domain of α_v_ and the βA domain of β_3_ were extracted from the crystal structure of the extracellular segment of integrin α_v_β_3_ (pdb code 1L5G) [26] and used as a (rigid) receptor for docking. In an initial test calculation the cyclic peptide ligand Cilengitide^®^ (cyclo-[RGDfN(Me)V]) [48] present in the X-ray structure [26] was redocked to the receptor in a “blind-docking” approach in which the search space covered almost the complete receptor surface (grid dimensions: 120 × 80 × 80, resolution 0.75 Å).

The input files for AUTODOCK were created with the help of “AutoDockTools” [52]. The genetic algorithm with local search option (GA-LS) as implemented in AUTODOCK was used to dock the flexible ligand. One hundred AUTODOCK jobs were started in parallel each performing 10 GA-LS runs, giving rise to 1000 individual GA-LS docking runs in total. For each GA-LS docking run 1,000,000 energy evaluations were performed. The Conformational Analysis Tools (CAT) program was used to merge the output data of the AUTODOCK runs, to perform the analysis of the entire dataset, and to organize the results in such a way that areas on the protein surface exhibiting a strong binding affinity could be easily visualized by using standard display programs.

In order to refine the docked structure of GSF a molecular dynamics (MD) simulation of the complex was performed by using AMBER [53]. The atomic partial charges and the topology files for GSF were prepared with *antechamber*. The final input files for *sander* were built with *tleap*. The MD simulations were run at 300 K in explicit water, by using periodic boundary conditions and following established standard protocols.

### Cell lines and culture conditions

The human melanoma cell lines WM-115 and WM-266-4 were obtained from the American Type Culture Collection (Manassas, VA). The WM-115 line was derived from a primary tumor and the WM-266-4 line from a cutaneous metastasis of the same patient. Both lines were maintained in Eagle’s minimum essential medium (MEM) with Earle’s salts (Biochrom, Berlin, Germany), 2 mM *L*-glutamine (PAA, Cölbe, Germany), 1 mM sodium pyruvate (Biochrom, Berlin, Germany), 0.1 mM nonessential amino acids (Biochrom, Berlin, Germany), 1.5 g/L NaHCO_3_ (Biochrom, Berlin, Germany) and 10% fetal bovine serum (FBS) (PAN Biotech, Aidenbach, Germany). Both lines were grown in a 5% CO_2_ atmosphere at temperatures of 34.5 °C (WM-115) or 37 °C (WM-266-4). Cells were passaged once a week by using 0.05% trypsin, 0.02% EDTA in PBS (PAA, Cölbe, Germany) to detach cells.

HBMEC-60 (retrovirally immortalized human-bone-marrow-derived endothelial) cells, kindly provided by Dr. E. van der Schoot (Sanguin, Amsterdam, The Netherlands) and originally described by Rood et al. [42], were grown in endothelial-specific culture medium (endothelial cell basal medium, PromoCell, Heidelberg, Germany), supplemented with 20% (v/v) FBS (Biochrom, Berlin, Germany), 1 μg/mL hydrocortisone, 0.1 ng/mL human epidermal growth factor and 1ng/mL human basal fibroblast growth factor, as recommended by the manufacturer. Cells used for the assays described below were mycoplasm free as verified by DAPI-staining of DNA and a PCR based mycoplasm test (Venor GeM-OneStep, Minerva Biolabs, Berlin, Germany).

### Cytotoxicity test

Cytotoxicity of GSF was tested using the sulforhodamine B (SRB) assay. Cells (1.3 × 10^4^/ well) were seeded into a 96-well plate and after 24 h incubation 2.5 to 10 mM of saccharide mimetics were added to the medium. After 24, 48 or 72 h the medium was gently removed and cells were fixed for 5 min at −20 °C with MeOH/HOAc, 95:5. After being washed three times with H_2_O and dried, the cells were stained with 0.4% SRB in 1% HOAc for 30 min. Wells were washed three times with 1% HOAc before the bound dye was dissolved with 10 mM Tris (pH 10.5). The absorbance at 546 nm was measured by using a microplate reader (µ-Quant, BIO-TEK Instruments Inc., Winooski, VT). The means and SD of quadruplicates were calculated.

#### Experimental conditions for human melanoma cells

**Adhesion assay:** To coat flexible 96-well plates (polyvinyl chloride (PVC), Falcon, Becton-Dickinson, Heidelberg, Germany) with human plasma ECM proteins, 0.5 µg human fibronectin (Invitrogen Karlsruhe, Germany) or 0.5 µg human fibrinogen (Calbiochem, Schwalbach, Germany) dissolved in 50 µL H_2_O was added to the wells and incubated overnight at 4 °C. Nonspecific binding sites were blocked with 200 µL of 1% bovine serum albumin (BSA) solution in phosphate-buffered saline (PBS). To control for nonspecific adherence to the PVC surface, cell adhesion was also measured on noncoated PVC plates.

Nearly confluent monolayers of WM-115 cells (48 h growth) were labeled with [methyl-^3^H]-thymidine (spec. activity 1.16–31.5 TBq/mmol, Hartmann Analytic, Braunschweig, Germany), 1.48 MBq/10^6^ cells/mL and incubated for 16 h at their respective temperatures. Cells were detached with 0.05% EDTA and washed three times in assay-medium (serum-free MEM-medium with Earle’s salts containing 0.25 mM MnCl_2_ and 0.1% BSA). Test compounds (the compounds described above, and methyl-β-D-galactose (Fluka, Taufkirchen, Germany), the peptides GRGDSP (Calbiochem, Darmstadt, Germany) or EILDV (synthesized by R. Pipkorn, DKFZ) were dissolved in assay medium and the cells were incubated therein for 1 h. For the adhesion assays on PVC, the assay medium contained no BSA. To each coated well 5 × 10^4^ cells were added, and after 1 h nonadherent cells were removed by three consecutive washing steps with PBS. Wells were cut out and transferred into scintillation vials, and then 5 mL scintillation cocktail (Ultima Gold, PerkinElmer, Boston, MA) was added and [^3^H] quantified by using a scintillation-counter (TriCarb 2200CA, Packard, Downers Grove, IL). Attached cells were quantified with a standard curve, which was performed for each assay, relating the cell number to [^3^H]-radioactivity. The number of adherent cells relative to the 5 × 10^4^ cells/well were calculated and related to the control incubations without test compounds. Standard deviations were calculated according to Bishop [54], taking the SD of the control into account.

**Cell migration (wound healing) assay:** Intact cell monolayers of WM-115 or WM-266-4 cells in 12-well plates (Becton Dickinson, Heidelberg, Germany) were wounded with a 100 µL pipette tip and washed three times with serum-free medium. Complete medium containing GSF was added to the wells and cell migration into the wound was observed by microscopy after 2, 4, 8 and 24 h and compared with migration in medium without GSF.

**Matrix metalloprotease (MMP) assays:** Zymographic analyses. Cells grown for 48 h were treated with GSF for 24 h in serum-free medium. Medium was removed and centrifuged for 8 min at 300*g*. Samples containing equal amounts of protein (determined by Lowry’s method) were separated under nonreducing conditions in a 7.5% sodium dodecyl sulfate-polyacrylamide gel (SDS-PAGE) polymerized together with 0.1% gelatin. After electrophoresis, gels were washed twice in 2.5% Triton X-100 and four times in H_2_O before overnight incubation in gelatinase buffer (0.02% Brij 35, Tris-HCl 50 mM, NaCl 150 mM, CaCl_2_ 10 mM, pH 7.6). Gelatinolytic activity was visualized by Coomassie-blue staining.

Western blot of MMP-2. Samples were prepared as described for zymography and separated in a 7.5% polyacrylamide gel under reducing conditions. Proteins were electroblotted onto nitrocellulose sheets (Schleicher & Schuell, Dassel, Germany) and the blots blocked for 30 min with 5% milk powder in PBS. Incubation with the primary mouse anti MMP-2 antibody (MAB13431, Chemicon, Temecula, Canada) in PBS with 0.5% human serum albumin was carried out overnight. After washing with PBS containing 0.1% tween-20 membranes were incubated for 4 h at rt with a rabbit anti-mouse alkaline phosphatase conjugated antibody (Dako, Glostrup, Denmark) in PBS with 0.5% human serum albumin. After additional washes the binding of the secondary antibody was visualized by BCIP/NBT-Blue (Sigma, Taufkirchen, Germany) as substrate.

#### Experimental conditions for human endothelial cell assays

**Adhesion assay:** For endothelial cell adhesion assays we used 96-well microtiter plates precoated either with laminin, fibronectin (both supplied by Becton Dickinson, Heidelberg, Germany) or collagen I (supplied by Greiner Bioscience, Frickenhausen, Germany). Cells were pre-incubated with the respective mimetic compounds in the concentrations indicated, for 30 min on ice. Prior to use, microtiter plates were washed with phosphate-buffered saline (PBS), then 4 × 10^4^ cells/100 μL culture medium without FBS or growth factors, and with or without mimetic compounds, were added to each well and cultivated for 30 min at 37 °C in a 5% CO_2_ atmosphere. Subsequently, the culture medium was decanted, and then the cells were washed twice in PBS and adherent cells fixed in 4% formaldehyde (v/v) in PBS for 5 min at rt. Subsequently, the fixation solution was removed, the plates were air dried and washed with 0.01 M borate, and the cells were stained in 1% methylene blue dissolved in 0.01 M borate for 10 min at rt. The staining solution was removed, and the cells were washed in water. The plates were air dried again and incubated with 200 μL/well of extraction buffer (0.1 M HCl/EtOH, 1:1 (v/v)) for 30 min at rt. Absorption was measured in an ELISA reader at 620 nm to quantify adherent cells. For certain experiments, cells were stimulated with 40 ng/mL human recombinant tumor necrosis factor (TNF) (PromoCell) 24 h prior to the test. Experiments were performed with six replicates.

**Migration assay:** In an endothelial migration assay, polycarbonate transwells (8 μm pore size, Corning Costar) were coated on the lower side with laminin, fibronectin or collagen type I (10 μg/mL in PBS each for 1 h at 37 °C) and were inserted into 24-well plates. Endothelial cells were plated at a density of 4 × 10^5^ cells/mL (100 μL/insert) into transwells in endothelial medium as described above, with or without 40 ng/mL TNF or 20 ng/mL VEGF and mimetic compounds, as indicated. After incubation for 30 min at 37 °C nonadherent cells were removed, and cells on the transwells were washed with PBS and fixed in 4% paraformaldehyde for 10 min at rt, washed again and stained in the dark with Hoechst 33342 DNA dye (Invitrogen), washed twice with PBS at rt and then stored at 4 °C until photographical documentation of the microscopy image and further counting of adherent stained cells.

**Matrigel in vitro angiogenesis assay:** The assay was performed as previously described [43]. Cells were preincubated with either 40 ng/mL TNF or 20 ng/mL VEGF for 24 h at 37 °C, before the cells (4 × 10^4^ cells/well in 300 μL medium) were added to 48-well plates coated with matrigel. The cells on matrigel plates were incubated overnight, with or without test compounds, in concentrations as indicated, at 37 °C, before the cellular networks were documented photographically and the networks quantified by a computational image-evaluation program.

**Tubing in vitro angiogenesis assay:** The cell preparation kit of TCS (Buckingham, UK) was used to perform the endothelial tubule formation assay. Cocultures of cells consisting of fibroblasts and human umbilical cord vein endothelial cells (HUVEC) were incubated in 24-well plates. On day 1, VEGF (20 ng/mL) and the test compounds in concentrations as indicated were added to the cell culture. The growth medium including growth factors and test substances was changed at days 4 and 7 of cell culture. At day 9 the medium was removed from the cell culture, and the cells were washed and fixed in 70% EtOH (v/v) for 30 min at rt, followed by a washing step and incubation in MeOH/30% H_2_O_2,_ 40:1 (v/v) for 10 min at rt. The cells were washed, then incubated with a monoclonal antibody against the CD31 antigen (Dako, Hamburg, Germany), which is specifically expressed on endothelial cells. They were then diluted 1:20 for 30 min, and, after a washing step, incubated with a secondary goat anti-mouse IgG antibody coupled to biotin (Dako) for 20 min, followed by a washing step and incubation with streptavidin coupled to horseradish peroxidase for 20 min. Antibody reactivity was visualized by adding AEC (3-amino-9-ethylcarbazole) chromogen substrate (Dako) to the cells for 14 min in the dark. Enzymatic reaction was stopped by washing with water. Wells were sealed with mounting medium and microscopic quantitative analysis of tube formation was performed with the software Angiosys 1.0, TCS (Cellworks). Statistical analyses were performed with GraphPad PRISM version 5.
